# Applicability of combined high-frequency and contrast-enhanced ultrasound in finger extensor tendon injuries: three case reports

**DOI:** 10.1186/s13089-024-00376-3

**Published:** 2024-07-17

**Authors:** Wenying Wang, Li Peng, Lian He, Yan Chen, Mingshan Jiang, Xue Luo, Guoqiang Gao

**Affiliations:** Department of Ultrasound, West China Longquan Hospital Sichuan University (The First People’s Hospital Of Longquanyi District), Chengdu, 610100 China

**Keywords:** Contrast enhanced ultrasound, High-frequency ultrasound, Extensor tendon of finger, Injury classification, Preoperative diagnosis

## Abstract

**Background:**

By combining high-frequency and contrast-enhanced ultrasound (CEUS), the position of the severed end of a finger extensor tendon injury and the injury classification can be determined as part of a comprehensive preoperative evaluation in clinical practice. However, there have been no reports of high-frequency ultrasound combined with CEUS for the preoperative diagnosis of human finger extensor tendon injury.

**Cases presentation:**

One case of complete rupture of the extensor tendon was diagnosed by ultrasound, which was completely consistent with the surgery; one case of incomplete rupture was ultimately confirmed clinically; and one case of distal phalangeal bone base avulsion fracture with tendon contusion and missed diagnosis on the first radiographic examination was confirmed by follow-up radiographic examination.

**Conclusions:**

Different types of finger extensor tendon injuries exhibit distinctive contrast-enhanced ultrasonography findings. Combined high-frequency and contrast-enhanced ultrasound can accurately locate the position of the severed end of the finger extensor tendon injury before surgery while observing the contrast agent filling area to clarify injury classification, providing a reliable imaging basis for clinical practice and ultimately developing personalized diagnosis and treatment plans for patients to ensure minimal trauma and pain, as well as optimal treatment effects.

## Background

An intact finger tendon is the foundation that ensures the completion of fine hand movements such as flexion, extension, clenching, and pinching. Finger tendon injuries can seriously affect a patient’s daily life and ability to work and are common in clinical practice. They are mostly caused by sudden external forces on the fingers. In addition, intrathecal injections of fluoroquinolone drugs [1], rheumatoid arthritis [2], and gout can cause tendon rupture due to pathological changes. Tendon injuries can be categorized as contusions, incomplete ruptures, and complete ruptures. Owing to the specific anatomy of the finger extensor tendon apparatus, rupture of the finger extensor tendons can be divided into incomplete tendon rupture, complete tendon rupture, and bone avulsion (i.e., avulsion fracture of the distal phalanx) [3]. Clinicians require information such as injury location, classification, distance between ruptured ends, and blood supply to the damaged tendons, to determine treatment plans.

The application of high-frequency ultrasound in muscles and bones has become established, well-known, and accepted by a large number of clinicians. In 2011, the European Federation of Medical and Biological Ultrasound Societies updated the applications of contrast-enhanced ultrasound (CEUS) for musculoskeletal diseases in the CEUS clinical practice guidelines and recommendations [4]. Domestic and foreign scholars have reported the application of CEUS in tendon pathology, such as in the rotator cuff [5], supraspinatus [6–8], and Achilles [9] tendons. However, there have been no reports of high-frequency ultrasound combined with CEUS for the preoperative diagnosis of human finger extensor tendon injury. This study was approved by the ethics committee of our hospital. Written informed consent was obtained from the patient for publication of this case report and accompanying images.

Instrumentation: two devices were used; the EPIQ5 color ultrasound device (Philips, Eindhoven, Netherlands), with a 18-4 MHz high-frequency linear array probe; and the Resona8 color ultrasound diagnostic instrument (Mindray, Shenzhen, China), with a 2.5–9.0 MHz high-frequency linear array probe.

Materials: a vial (59 mg) of SonoVue microbubble contrast agent (Bracco, Milan, Italy) was diluted with 5 mL saline and shaken well to form a microbubble suspension.

High-frequency ultrasound examination: the participants sat on the opposite side of the examination bed and placed their hands on the examination bed. With the palm flat downwards, an object (such as a roll of paper or box) was placed under the palm, and the coupling acoustic pad was placed on the back of the hand. For the longitudinal scanning, the probe was placed parallel to the longitudinal axis of the tendon. The probe was moved gradually along the tendon from the distal phalangeal attachment to the musculotendinous junction. We checked for tendon echogenicity (continuous, reduced, or enhanced), localized thickening or thinning, fluid accumulation around the tendon sheath or tendon, phalangeal bone cortex continuity, and for the presence of bone fragments. Dynamic evaluation of tendon activity at the distal end of flexion and extension of the finger joint was performed together with observation of the traction sensation in the proximal tendon.

CEUS examination method: The examination position was the same as that for a simple, high-frequency ultrasound examination. In the CEUS double-contrast mode, 5 mL of the suspension was extracted and rapidly injected through the cubital vein and finally flushed with 5 mL of physiological saline. A real-time dual-contrast mode observation was conducted and the enhancement time of the tendon target site and the location and range of contrast agent infusion were recorded.

Injuries were classified using high-frequency ultrasound and CEUS imaging features and compared with clinical surgical results and X-ray examinations.

### Cases presentation

**Fig. 1:**
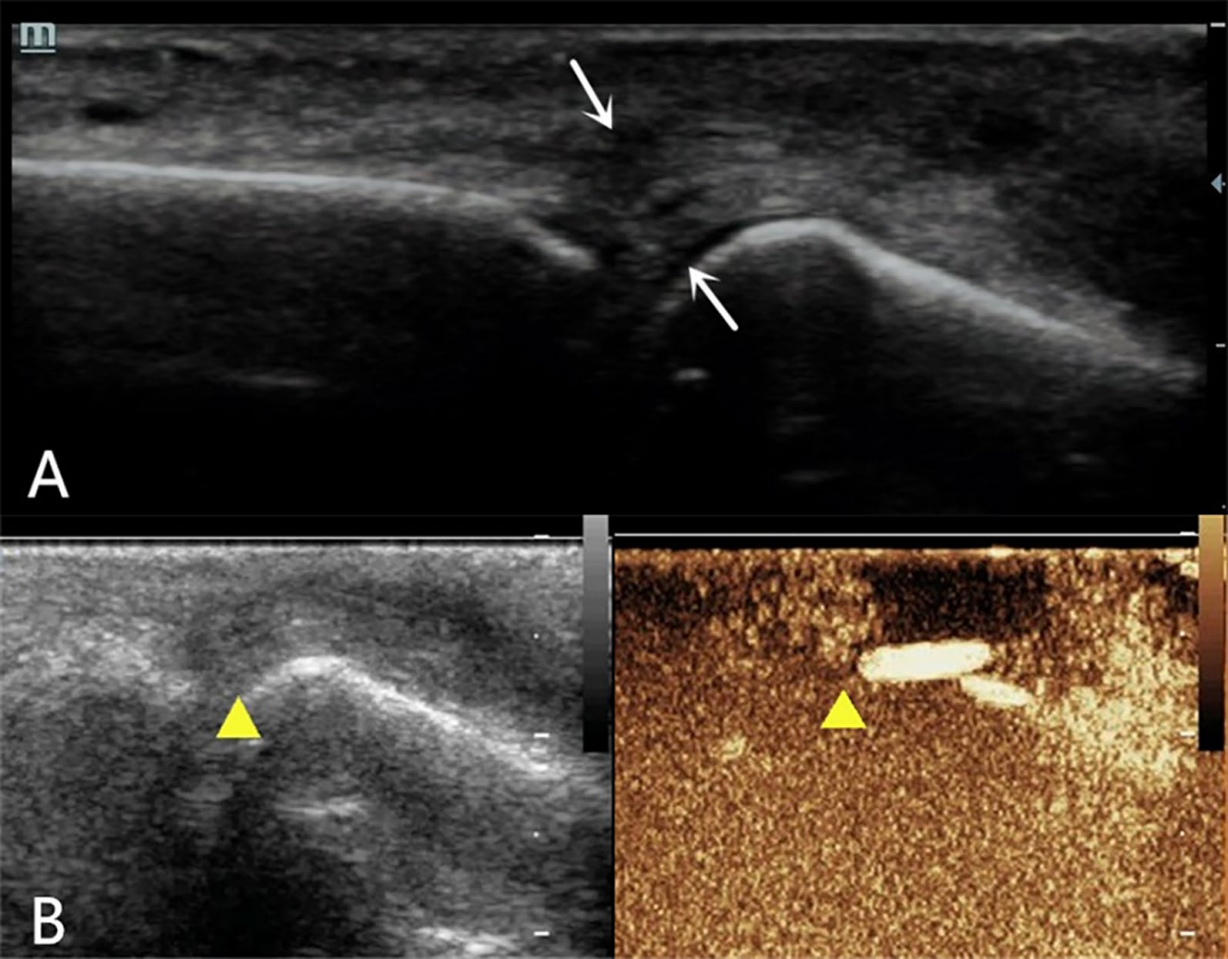
Ultrasound examination of a 52-year-old male with right fourth finger extensor tendon injury. **A** Two-dimensional ultrasound showed that the horizontal extensor tendon of metacarpophalangeal joint was thickened, with reduced echogenicity and a visible crease (indicated by white arrow) **B**. CEUS shows an “up and down through”  type filling perfusion of contrast agent inside the tendon (indicated by a solid yellow triangle)

**Fig. 2:**
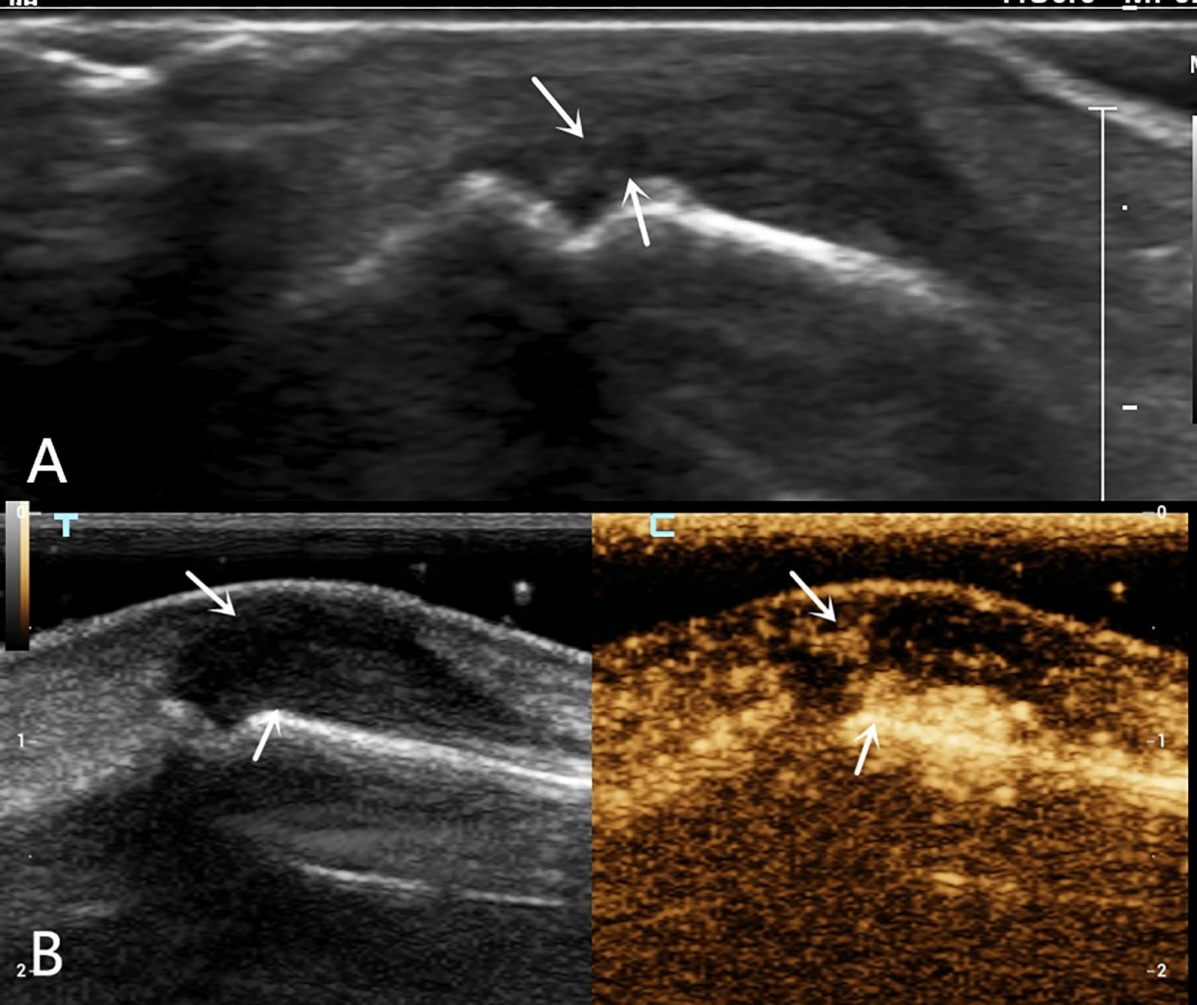
Ultrasound examination of a 53-year-old female with a second left finger tendon injury. **A**. Two-dimensional ultrasound shows thickening and reduced echogenicity of the extensor tendon at the level of the metacarpophalangeal joint, with suspicious fissure-like hypoechogenicity within the tendon (indicated by a white arrow). **B**. CEUS shows “triangular” filling perfusion in the superficial and deep layers of the tendon (indicated by a white arrow)

**Fig. 3:**
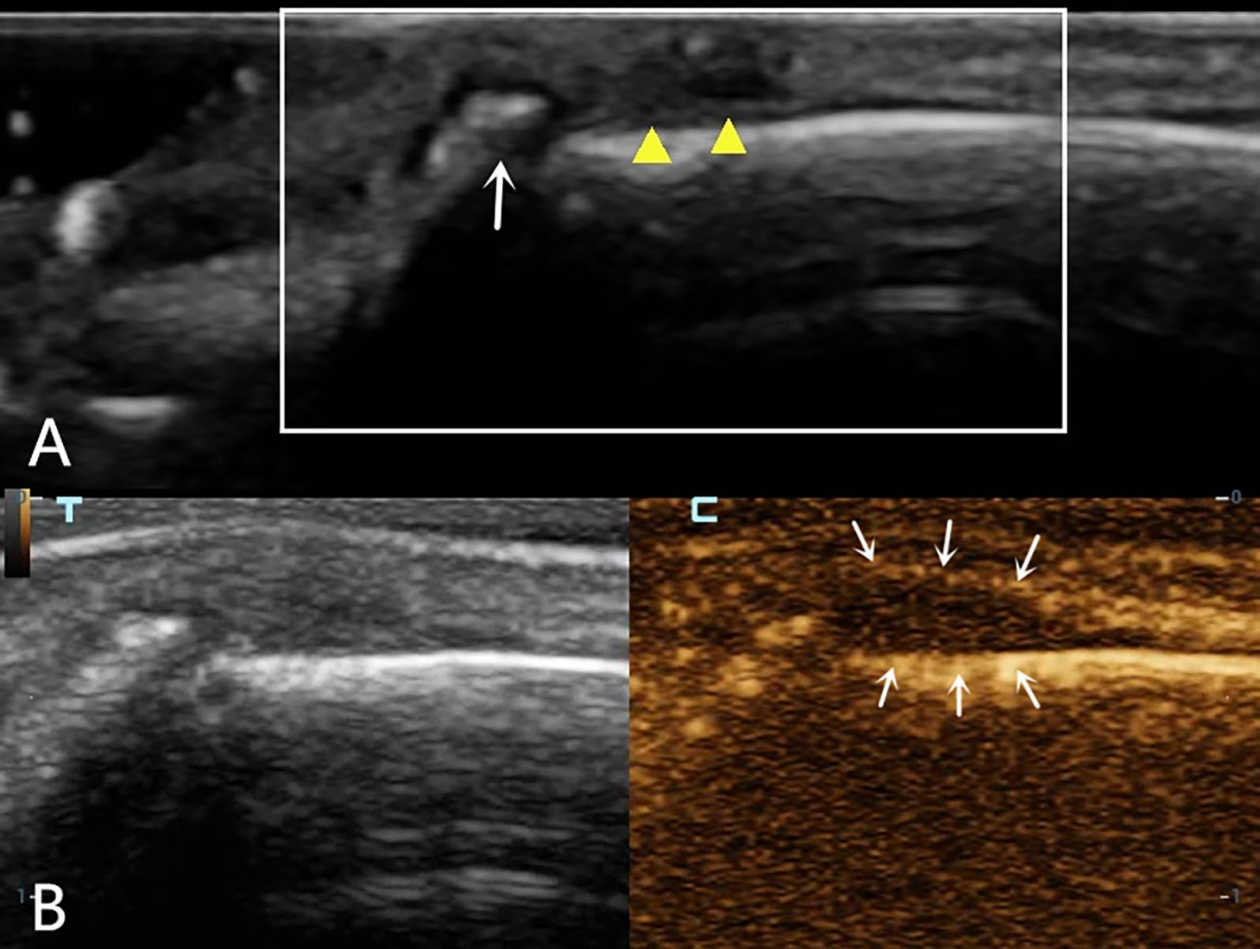
Ultrasound examination of a 34-year-old male with a left fourth finger extensor tendon injury. **A**. Two-dimensional ultrasound shows thickening and reduced echogenicity of the extensor tendon at the level of the distal interphalangeal joint (indicated by a solid yellow triangle). Avulsion fracture fragments with strong echogenicity can be seen at the base of the distal phalange (white arrow). **B**. CEUS showing no contrast agent filling inside the extensor tendon (indicated by a white arrow)

1. A 52-year-old male patient scratched the back of his right hand while cleaning a range hood 20 days before the examination and presented with restricted extension of the fourth finger of his right hand. High-frequency ultrasound examination showed that the extensor tendon of the right fourth finger was thickened at the level of the metacarpophalangeal joint, with reduced echogenicity and visible creases in the tendon fibers (Fig. 1A). When the fingertip was passively flexed and extended, the proximal tendon exhibited poor mobility. At this point, a tendon injury was diagnosed using two-dimensional ultrasound; however, it remained unclear whether it was a complete or partial injury. To confirm the diagnosis, the physician decided to perform a CEUS evaluation, which showed early and high enhancement in the area of low tendon echogenicity, showing an “up and down through”-type enhancement on both sides (Fig. 1B). The final diagnosis was a complete rupture of the extensor tendon. The patient underwent a digital extensor tendon suture surgery. During surgery, the tendon injury and location of the ruptured end were found to be completely consistent with the ultrasonic examination results. The surgery proceeded smoothly, with 5 mL of intraoperative bleeding. The postoperative incision healed well with good finger circulation and sensation. The movement of the fourth finger of the right hand improved significantly after surgery.
2. A 53-year-old female patient had limited extension of the second finger of her left hand 6 days after being stabbed by a knife. High-frequency ultrasound examination showed that the horizontal extensor tendon of the left second finger was significantly thickened, with reduced and uneven echogenicity. Both the tendon fibers and surrounding tissues were hypoechoic (Fig. 2A), and there appeared to be a fissure-like hypoechogenicity inside the tendon; it was therefore unclear whether there was a single pure contusion swelling or a partial rupture. CEUS was then performed and showed early and high enhancement in the target area of the extensor tendon, triangular enhancement in the superficial and deep torn areas, and a linear, non-enhanced area in the middle (Fig. 2B). An incomplete rupture of the extensor tendon of the second finger was ultimately diagnosed. The orthopedic surgeon decided not to perform surgery and developed a rehabilitation exercise plan. After 5 months, the patient's second finger extension function improved significantly, and there was slight pain when applying force.
3. A 34-year-old male patient was unable to straighten the fourth finger of the left hand for 2 days after trauma. On the first day after the patient's arrival, radiographic examination showed no obvious bone abnormalities. On the second day, high-frequency ultrasonography revealed a discontinuity in the cortical bone of the distal phalanx, with strong echogenic bone fragments that were torn off and attached to the extensor digitorum tendon. The extensor tendon had become thicker and showed reduced echogenicity, with a hypoechoic area visible within the tendon (Fig. 3A). Two-dimensional ultrasound was used to diagnose an avulsion fracture of the distal phalanx of the left fourth finger, but an incomplete rupture of the extensor tendon could not be ruled out. The CEUS examination showed no significant contrast agent perfusion inside the extensor tendon (Fig. 3B), leading to a final ultrasound diagnosis of avulsion fracture of the distal phalanx of the left fourth finger with a tendon contusion. According to the ultrasound examination results, the orthopedic doctor arranged for a follow-up X-ray examination, and the results showed a linear translucent shadow on the dorsal side of the distal phalanx of the left fourth finger, indicating a possible fracture. The orthopedic surgeon performed internal fixation surgery on the fourth finger, and the extension function of the fourth finger improved significantly after surgery.

## Conclusions

Early identification and classification of tendon injuries are crucial. Timely and effective restoration of tendon length, continuity, and reconstruction of blood supply to the injured tendon can help maximize finger function recovery. However, owing to factors such as local tendon edema complicated by hematoma, local soft tissue contusion during tendon tears, and complex ultrasound manifestations at the tear site often affect observation. Most of the tendon tissue was ruptured, with only a small number of tendon fibers attached to the bottom of the phalanges, and the sense of traction was not obvious. A single high-frequency ultrasound cannot easily distinguish partial from complete tendon rupture, and misdiagnosis is easy to occur, leading to unnecessary surgery, causing patients to suffer avoidable postoperative complications, and increasing their pain and burden.

Different subtypes of finger extensor tendon injuries have characteristic CEUS findings. Complete rupture is characterized by “up and down through”-type filling perfusion, incomplete rupture is characterized by “triangular” filling perfusion in the tear area, and contusion has no obvious contrast agent filling perfusion. Combined high-frequency and CEUS can accurately locate the severed end of finger extensor tendon injury before surgery, classify the injury accurately, enable physicians to comprehensively understand the condition of the tendon injury, and develop personalized diagnosis and treatment plans that minimize trauma, pain, treatment time, and optimize treatment effects.

## Data Availability

All scans obtained are available via the corresponding author.

## Abbreviation

CEUS: Contrast-enhanced ultrasonography
